# Characterization of the Muscle Electrical Properties in Low Back Pain Patients by Electrical Impedance Myography

**DOI:** 10.1371/journal.pone.0061639

**Published:** 2013-04-22

**Authors:** Congo Tak-Shing Ching, Yueh-Chi Chen, Li-Hua Lu, Peiyuan F. Hsieh, Chin-Sung Hsiao, Tai-Ping Sun, Hsiu-Li Shieh, Kang-Ming Chang

**Affiliations:** 1 Department of Electrical Engineering, National Chi Nan University, Nantou, Taiwan, ROC; 2 Department of Applied Materials and Optoelectronic Engineering, National Chi Nan University, Nantou, Taiwan, ROC; 3 Department of Photonics and Communication Engineering, Asia University, Taichung County, Taiwan, ROC; 4 School of Physical Therapy, Chung Shan Medical University, Taichung, Taiwan, ROC; 5 Physical Therapy Room, Chung Shan Medical University Hospital, Taichung, Taiwan, ROC; 6 Division of Neurology, Taichung Veterans General Hospital, Taichung, Taiwan, ROC; 7 Department of Applied Chemistry, National Chi Nan University, Nantou, Taiwan, ROC; 8 Graduate Institute of Clinical Medical Science, China Medical University, Taichung, Taiwan, ROC; University of California, San Diego, United States of America

## Abstract

**Objectives:**

This study aims to investigate the electrical properties of lumbar paraspinal muscles (LPM) of patients with acute lower back pain (LBP) and to study a new approach, namely Electrical Impedance Myography (EIM), for reliable, low-cost, non-invasive, and real-time assessment of muscle-strained acute LBP.

**Design:**

Patients with muscle-strained acute LBP (n = 30) are compared to a healthy reference group (n = 30). Electrical properties of LPM are studied.

**Background:**

EIM is a novel technique under development for the assessment of neuromuscular disease. Therefore, it is speculated that EIM can be employed for the assessment of muscle-strained acute LBP.

**Methods:**

Surface electrodes, in 2-electrode configurations, was used to measure the electrical properties of patient's and healthy subject's LPM at six different frequencies (0.02, 25.02, 50.02, 1000.02, 3000.02, and 5000.02 kHz), with the amplitude of the applied voltage limited to 200 mV. Parameters of impedance (Z), extracellular resistance (R_e_), intracellular resistance (R_i_), and the ratio of extracellular resistance to intracellular resistance (R_e_/R_i_) of LBP patient's and healthy subject's LPM were assessed to see if significant difference in values obtained in muscle-strained acute LBP patients existed.

**Results:**

Intraclass correlation coefficient (ICC) showed that all measurements (ICC>0.96 for all studying parameters: Z, R_e_, R_i_, and R_e_/R_i_) had good reliability and validity. Significant differences were found on Z between LBP patient's and healthy subject's LPM at all studying frequencies, with p<0.05 for all frequencies. It was also found that R_e_ (p<0.05) and R_e_/R_i_ (p<0.05) of LBP patient's LPM was significant smaller than that of healthy subjects while R_i_ (p<0.05) of LBP patient's LPM was significant greater than that of healthy subjects. No statistical significant difference was found between the left and right LPM of LBP patients and healthy subjects on the four studying parameters.

**Conclusion:**

EIM is a promising technique for assessing muscle-strained acute LBP.

## Introduction

Lower back pain (LBP) is common in the general population, affecting 60–80% of all adults at some point in their life [1], [2]. In the United States and United Kingdom between 70% and 80% of the adult population have suffered from LBP at some time and have contacted a general practitioner [3]–[5]. LBP is one of the most common symptoms reported by Taiwan people [6], [7]. The life and 12-month incidence rate of LBP among Taiwan people was 37.0% and 18.9%, respectively [6]. LBP is a common reason for patients to seek medical care. In Taiwan alone more than 2.14 million patients sought medical care for back pain in 1998 and that medical cost alone exceeded 3 billion New Taiwan Dollars [8]. The possible consequences of unmanaged LBP are well documented, e.g. depression [9], [10] and functional disability [11]. LBP is costly in terms of treatment, individual suffering and work absenteeism [12].

Several diagnostic methods are available to diagnose the cause of LBP so as to give an appropriate treatment: x-ray imaging, discography, computerized tomography, magnetic resonance imaging, electrodiagnostic procedures (electromyography, nerve conduction studies, and evoked potential studies), bone scans, and ultrasound imaging. However, most LBP mainly arises from the problem of low back muscles [13]–[17] and therefore some of these diagnostic methods are ineffective on diagnosing muscle-strained acute LBP. For example, x-ray imaging and computerized tomography are good for bone diagnosis but not ideal for the diagnosis of muscle problem. On the other hands, some of these diagnostic methods are either invasive or costly. For instance, discography and bone scans are invasive methods, as they involve the injection of a special contrast dye and a radioactive material respectively for diagnosis, and they are also not the methods for the diagnosis of muscle problem. Although magnetic resonance imaging is the best way to obtain a definitive diagnosis of muscle-strained acute LBP, it is costly. The drawbacks of electrodiagnostic procedures and ultrasound imaging are that they cannot give information about electrochemical processes and physiological changes in the muscle.

Electrical Impedance Myography (EIM) is a bioimpedance-based technique. It is the measurement of the bioimpedance signal, which is obtained by injecting low-level sinusoidal current in the tissue and measuring the voltage drop generated by the tissue impedance. Bioimpedance signal gives information about electrochemical processes in the tissue and can hence be used for characterizing the tissue or for monitoring physiological changes. EIM is well established. It has been used for investigating the physiological properties of muscles [18]–[24] and has been effective in the assessment of neuromuscular diseases [22]–[24]. The basic concept underlying the use of EIM as a clinical tool is that pathological muscle changes such as muscle fiber atrophy and loss, tissue edema, and fatty replacement alter impedance signatures in consistent ways. For example, Aaron et al. in 2006 [21] used EIM for the assessment of quadriceps and tibialis anterior muscles to the effects of normal aging and they found that there are muscle reductions, in terms of the spatially averaged phase angle, with increasing age. The definition of phase angle refers to the relationship between the two vector components, i.e. resistance and reactance, of impedance. Other works have been demonstrated that the phase angle decreases in generalized neuromuscular diseases including myopathy and motor neuron disease, as well as in more focal disorders, such as radiculopathy [22]–[24]. At present, no studies using EIM for the investigation of muscle-strained acute LBP are reported in the literature. Muscle changes which occur in muscle-strained acute LBP are expected to affect EIM outcome parameters much as they do in disease, including changes in the membrane properties and the composition of the intra-cellular and extra-cellular matrices. Earlier EIM studies have shown that its principal outcome variable, the spatially averaged phase angle, rapidly drops with increasing severity in generalized disorders such as inflammatory myositis and amyotropic lateral sclerosis [22], [24], [25]. Therefore, the aim of this study is to study the electrical properties of lumbar paraspinal muscles (LPM) of patients with LBP and to investigate the EIM approach for reliable, low-cost, non-invasive, and real-time assessment of muscle-strained acute LBP.

## Materials and Methods

Thirty patients (14 men and 16 women; age: 45±7 year; weight: 61±9 kg) diagnosed with muscle-strained acute LBP by rehabilitation medical doctor and another thirty healthy subjects (15 men and 15 women; age: 48±10 year; weight: 67±13 kg) without LBP in the past 6 months were recruited in this study. The study was approved by the Asia University Medical Research Ethics Committee. Each participant provided his/her written informed consent to participate in this study.

Four electrical properties of LPM were investigated: impedance (Z), extracellular resistance (R_e_), intracellular resistance (R_i_), and the ratio of extracellular resistance to intracellular resistance (R_e_/R_i_).

**Figure 1:**
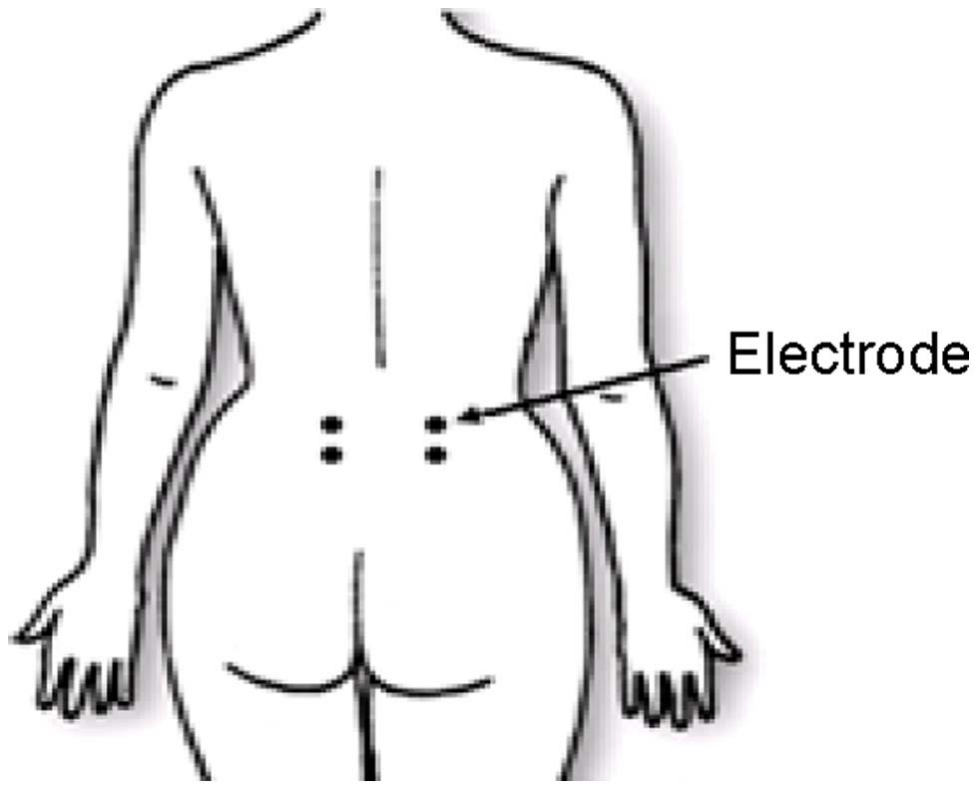
Position of electrodes. All electrodes are positioned along the LPM. The center of both distal electrodes is 5 cm below the fifth lumbar vertebra and the center of the two electrodes on each side was 5 cm apart.

EIM measurements of the LPM were made with LBP patients and healthy subjects lying on a bed in the prone position. Disposable surface electromyographic electrodes (3M red. dotTM 2258T Ag/AgCl) were used and positioned at a bipolar electrode configuration (Figure 1). In brief, two electrodes were positioned along the left LPM region of the LBP patients and healthy subjects, with the center of the distal electrode 5 cm below the fifth lumbar vertebra and the center of the two electrodes was 5 cm apart. Another two electrodes were positioned in the same manner at the right LPM region of the LBP patients and healthy subjects. Prior to the electrodes attachment, the areas of skin where the electrodes to be located were prepared by briskly rubbing the areas for 8 seconds with alcohol prep pads to remove dry skin, oils and other contaminants. The areas were then allowed to dry thoroughly.

### 1. Measurement procedures

Electrodes at each side (left and right side) were separately connected to an impedance analyzer (Precision Impedance Analyzer WK6420C, Wayne Kerr Electronics Ltd, United Kingdom) for each LBP patient and healthy subject measurement. At each side, EIM measurements were made at six frequencies (0.02, 25.02, 50.02, 1000.02, 3000.02 and 5000.02 kHz), with the amplitude of the applied voltage limited to 200 mV, and each EIM measurement took about 2 minutes to be finished. In all cases, three separate sets of measurement of each side were made in succession in order to check reliability of the EIM measurements. On the other hand, the results of these three separate sets of measurement were averaged for the between groups and within groups comparisons. Therefore, each patient/subject spent about 12 to 15 minutes for the whole experiment.

Before and after each LBP patient and healthy subject measurement, their lower back skin-surface temperatures (LBSSTemp) were measured using infrared thermometer (TemporalScanner™ 2000C, Exergen Corporation, USA). This is because that impedance of tissue varies with temperature [26]. And, this procedure is used to see whether the change of the tissue impedance is due to the effect of temperature differences.

### 2. Data Processing

**Figure 2:**
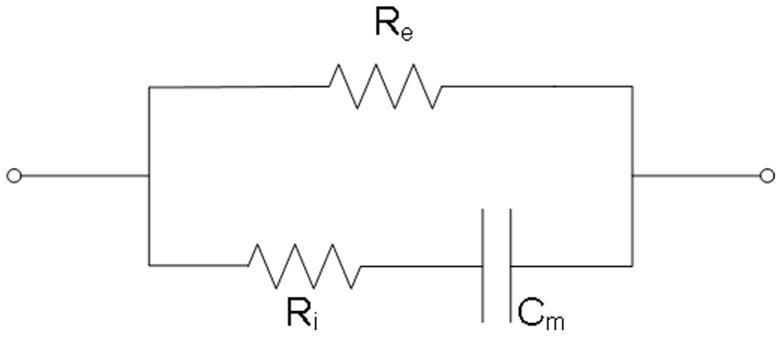
An equivalent circuit of LPM. R_e_ and R_i_ are the resistance of the extracellular and intracellular medium of the LPM. C_m_ is the capacitance of the cell membrane.

Yamamoto and Yamamoto suggested that a tissue can be electrically represented in impedance terms as a combination of a resistor placed in parallel with another resistor and a capacitor in series [27], an equivalent circuit of the impedance of the LPM can be established as shown in Figure 2, with impedance Z:
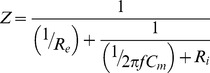
(1)where R_e_ and R_i_ are the resistance of the extracellular and intracellular medium of the LPM respectively, C_m_ is the capacitance of the cell membrane, and f is the frequency.

When the frequency approaches zero, Equation 1 can be rewritten:
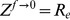
(2)where Z^f→0^ is the impedance of the LPM as frequency closed to zero (0.02 kHz in this study).

When the frequency approaches infinity, Equation 1 can be rewritten:
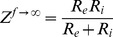
(3)where Z^f→∞^ is the impedance of the LPM as closed to infinity (5000.02 kHz in this study).

Electrical properties R_e_, R_i_, and R_e_/R_i_ of the LPM can thus be determined from Equations 2 and 3.

### 3. Statistical analysis

Intraclass correlation coefficient (ICC) was used to evaluate intrarater reliability (ICC 3,1) for the measurement of Z, R_e_, R_i_, and R_e_/R_i_. Paired-sample t test (within group) and independent-samples t test (between groups) were used to determine whether there were significant differences between LBP patient's and healthy subject's LPM for the studying parameters of Z, R_e_, R_i_, and R_e_/R_i_ at each frequency and their LBSSTemp. All statistical analyses were carried out using SPSS software with the level of statistical significance set at 0.05.

## Results

Thirty patients with muscle-strained acute LBP and thirty healthy subjects with no LBP in the past 6 months participated in this study. Their LBSSTemp before and after experiment were recorded and summarized in Table 1. No statistical significant difference was found on LBP patients' pre- and post-experimental LBSSTemp as well as on healthy subjects' pre- and post-experimental LBSSTemp. Also, no statistical significant difference was found between the pre-experimental LBSSTemp of LBP patients and that of healthy subjects as well as between the post-experimental LBSSTemp of LBP patients and that of healthy subjects.

**Table 1:** The lower back skin-surface temperature of LBP patients and healthy subjects before and after experiment.

	Lower Back Skin-Surface Temperature (°C)		bP-Value
	*Before Experiment*	*After Experiment*	
**LBP Patients**	36.7±0.2	36.6±0.2	>0.05
**Healthy Subjects**	36.5±0.2	36.5±0.2	>0.05
a **P-Value**	>0.05	>0.05	

a Independent-samples t test.

b Paired-sample t test.

ICC for the intrarater reliability (ICC 3,1) for the measurement of electrical parameters (Z, R_e_, R_i_, and R_e_/R_i_) of LPM were summarized in Table 2. All electrical parameter measurements for the LPM of LBP patients and healthy subjects had ICC value ranging from 0.96 to 0.99.

**Table 2:** Estimation of intrarater reliability (ICC 3,1) for the measurement of impedance (Z) extracellular resistance (R_e_), intracellular resistance (R_i_), and the ratio of extracellular resistance to intracellular resistance (R_e_/R_i_) of LPM.

	ICC 3,1			
	*Z*	*R_e_*	*R_i_*	*R_e_/R_i_*
**LBP Patients**	0.99	0.96	0.99	0.98
**Healthy Subjects**	0.99	0.98	0.98	0.99

**Figure 3:**
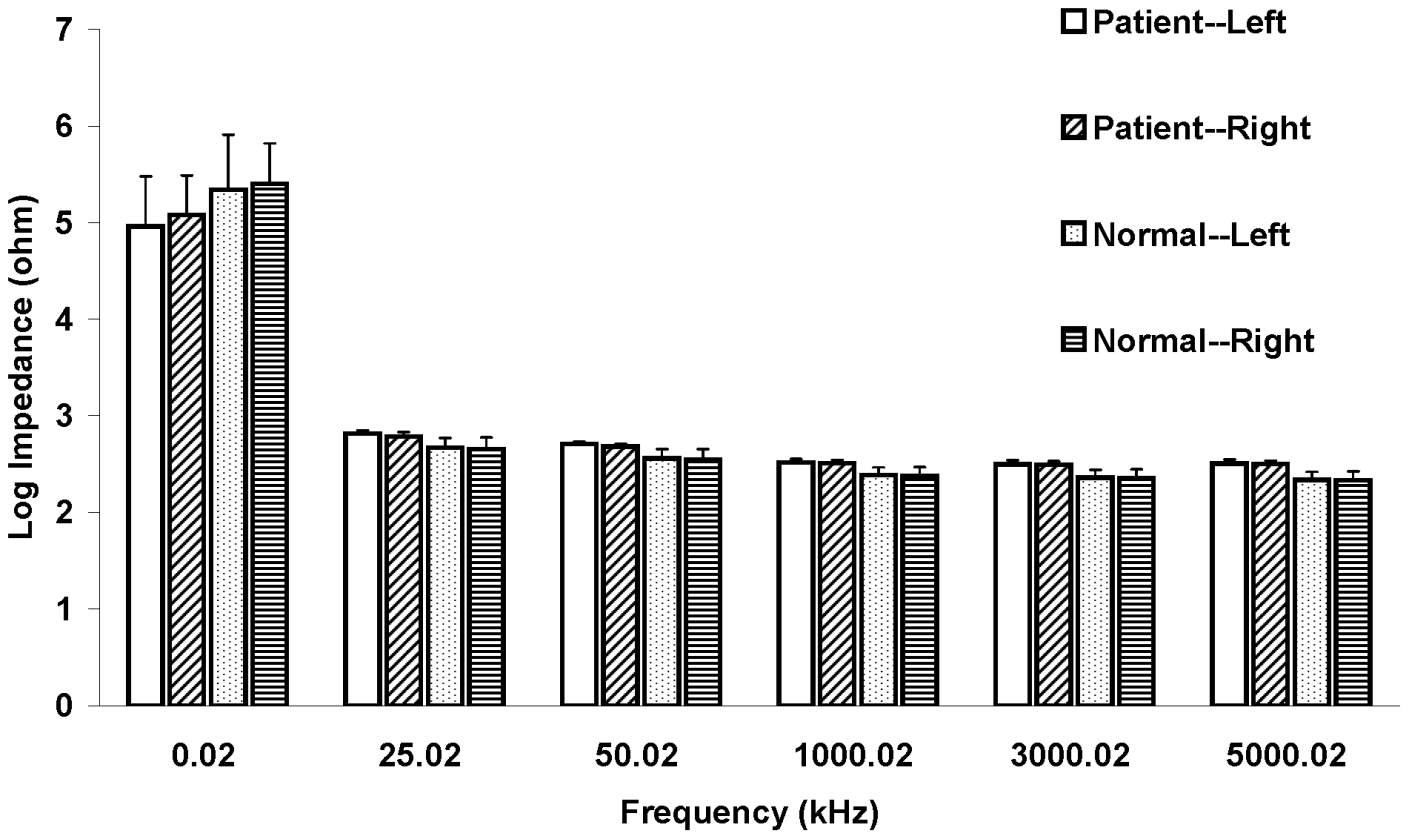
Impedance spectrum of LPM. Impedance (Z) of LPM measured at different frequencies (n = 30). Results were expressed as means and standard deviations. Z of LBP patient's LPM was found to be significantly smaller (p<0.05) than that of healthy subject's LPM at 0.02 kHz. However, Z of LBP patient's LPM was found to be significantly higher (p<0.05) than that of healthy subject's LPM at the remaining frequencies (25.02, 50.02, 1000.02, 3000.02 and 5000.02 kHz). No statistical significant difference was found between the left and right LPM of LBP patients and healthy subjects on Z.

**Figure 4:**
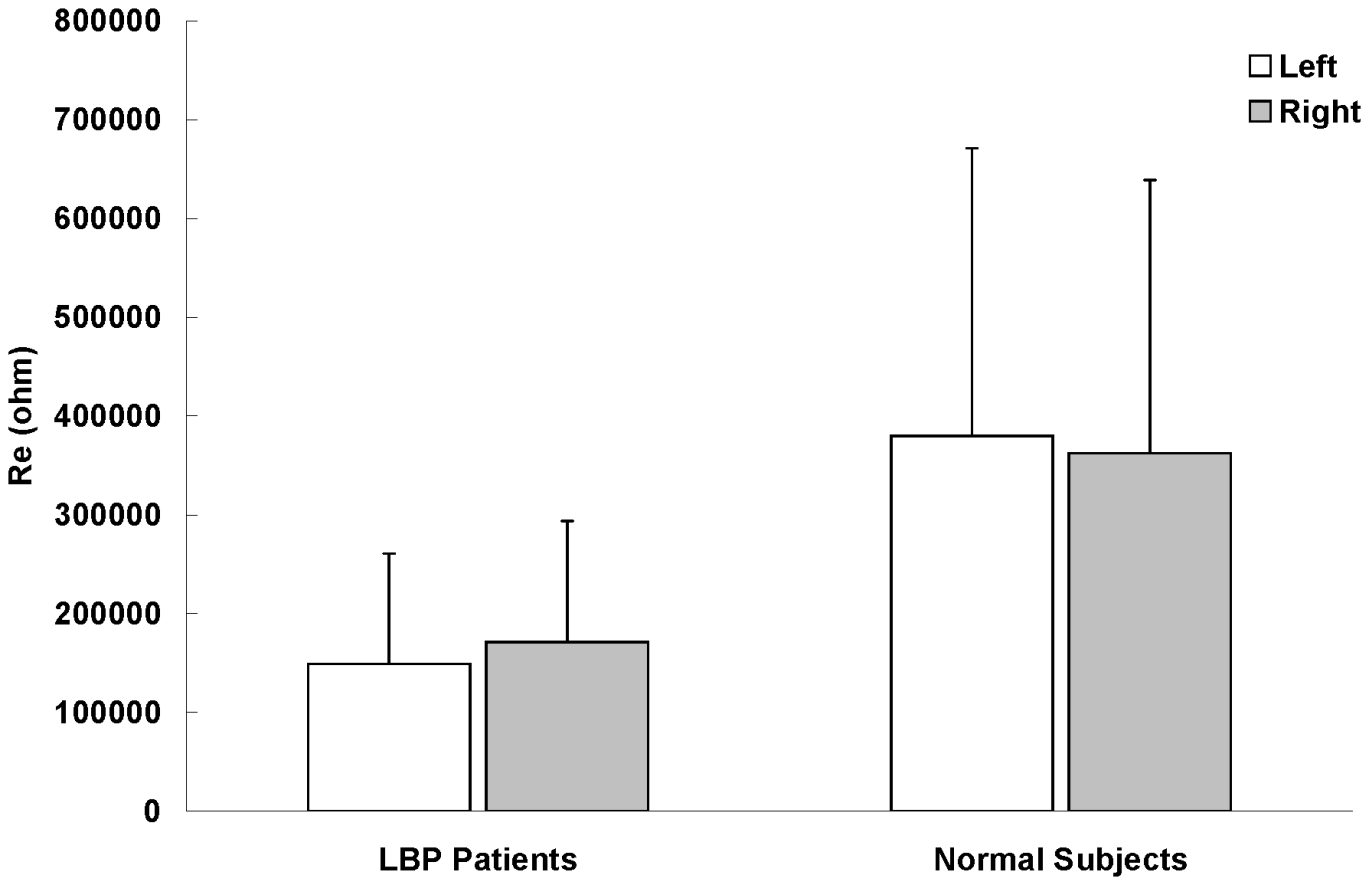
Extracellular resistance (R_e_) of LPM (n = 30). Results were expressed as means and standard deviations. R_e_ of LBP patient's LPM was found to be significant smaller (p<0.05) than that of healthy subjects. However, no statistical significant difference was found between the left and right LPM of LBP patients and healthy subjects on R_e_.

**Figure 5:**
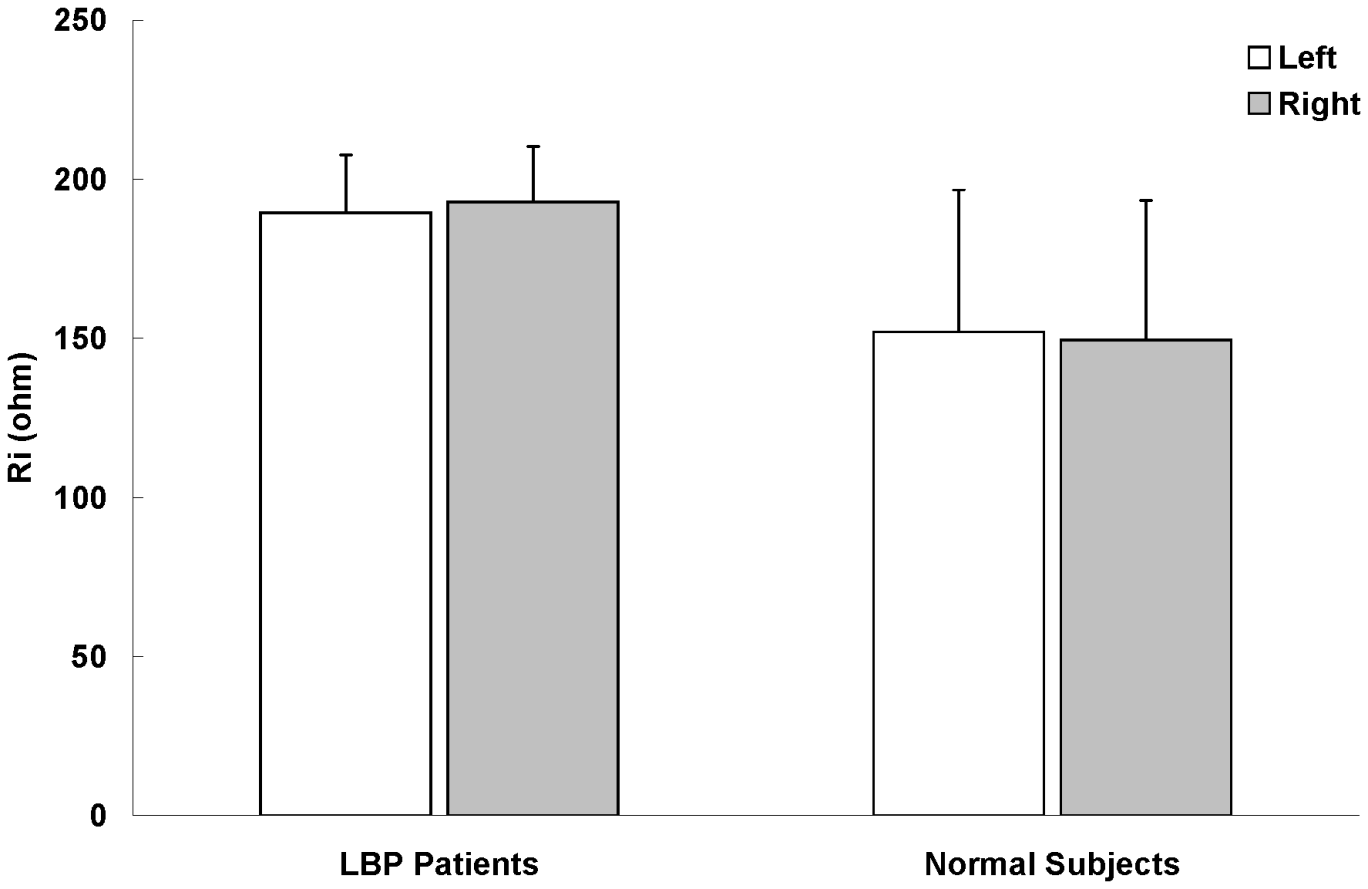
Intracellular resistance (R_i_) of LPM (n = 30). Results were expressed as means and standard deviations. R_i_ of LBP patient's LPM was found to be significant higher (p<0.05) than that of healthy subjects. However, no statistical significant difference was found between the left and right LPM of LBP patients and healthy subjects on R_i_.

**Figure 6:**
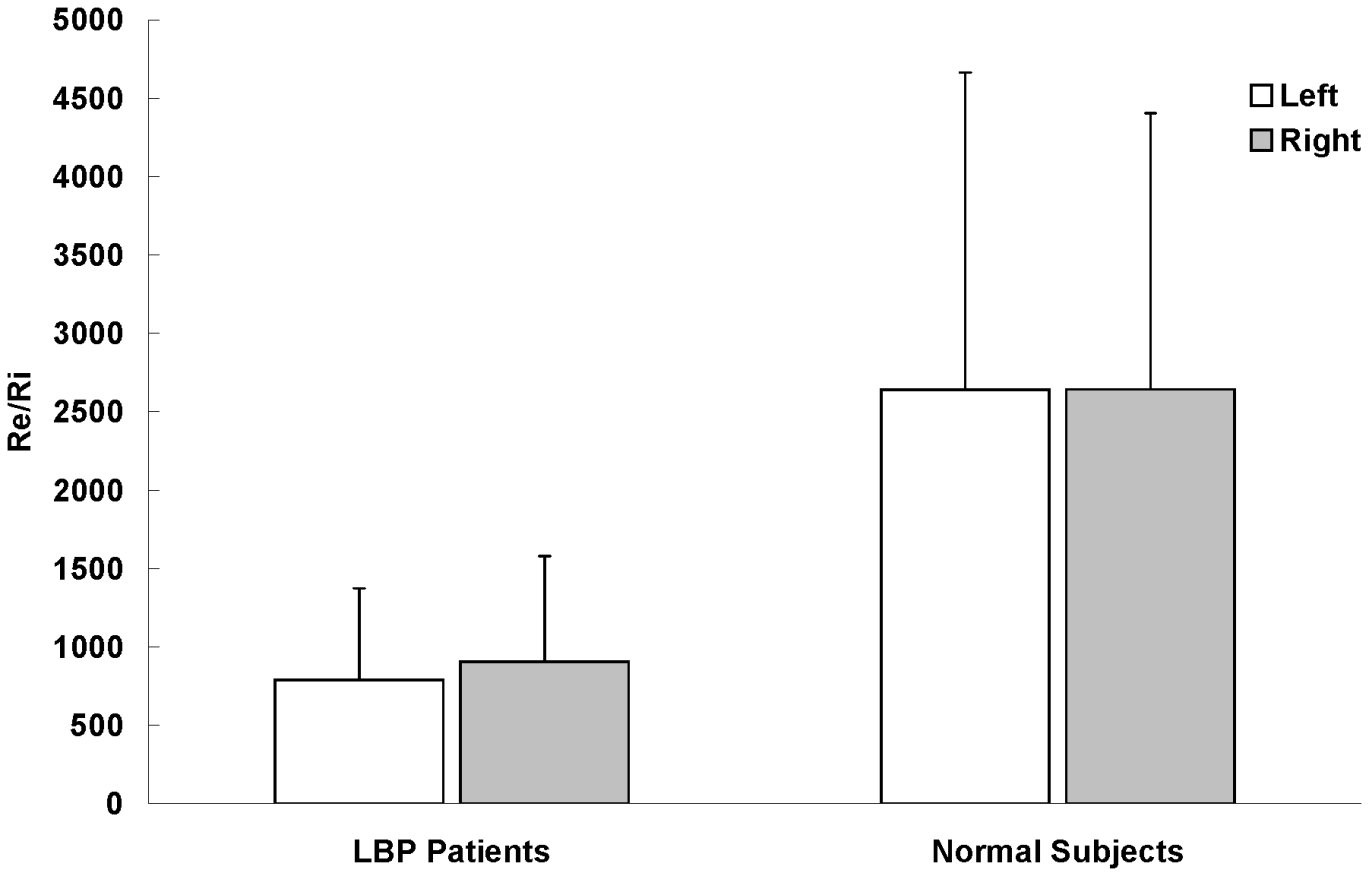
The ratio of extracellular resistance to intracellular resistance (R_e_/R_i_) of LPM (n = 30). Results were expressed as means and standard deviations. R_e_/R_i_ of LBP patient's LPM was found to be significant smaller (p<0.05) than that of healthy subjects. However, no statistical significant difference was found between the left and right LPM of LBP patients and healthy subjects on R_e_/R_i_.

The electrical properties (Z, R_e_, R_i_, and R_e_/R_i_) of LPM were summarized in Figures 3, 4, 5 and 6. Paired-sample t test was used to determine whether there were significant differences on the electrical properties of LPM between the left and right sides of LBP patients as well as between the left and right sides of healthy subjects. No statistical significant difference was found on the four studying parameters (Z, R_e_, R_i_, and R_e_/R_i_) of LPM between the left and right sides of LBP patients as well as of healthy subjects. Therefore, results from the left and right sides were averaged when comparing between the two study groups (i.e. the patient and healthy subject groups).

As shown in Figure 3, it was observed that Z of LBP patient's and healthy subject's LPM decreased as the measurement frequency increasing. Moreover, Z of LBP patient's LPM was found to be significantly (p<0.05, about 0.9-fold) smaller than that of healthy subject's LPM at 0.02 kHz. However, Z of LBP patient's LPM was found to be significantly (p<0.05, about 1.1-fold) higher than that of healthy subject's LPM at 25.02, 50.02, 1000.02, 3000.02 and 5000.02 kHz.

It was found that R_e_ of LPM of LBP patient was significantly smaller (p<0.05, about 0.4-fold) than that of healthy subjects (Figure 4). The R_e_/R_i_ ratio of LPM of LBP patient was also found to be significantly smaller (p<0.05, about 0.3-fold) than that of healthy subjects (Figure 6). However, R_i_ of LPM of LBP patient was found to be significantly greater (p<0.05, about 1.3-fold) than that of healthy subjects (Figure 5).

## Discussion

The LPM plays an important role as stabilizers and therefore has been a focus of active research. For example, in patients with LBP, medical imaging studies of the paraspinal muscles consistently show a decrease in cross-sectional areas [28], [29]. Therefore, this study focuses on the LPM in order to study the electrical properties difference between LBP patients and healthy subjects.

In this study, before and after each participator (LBP patient and healthy subject) measurement, his/her LBSSTemp was measured. This is because that impedance of tissue varies with temperature [26]. Some participators were found to have 0.1°C change in their LBSSTemp whereas the rest of the participators had no change in their LBSSTemp. Participators' actual skin-surface temperature change might be the source of such temperature changes. Another possible reason for such temperature changes might be due to the instrumentation error during measurement as the infrared thermometer has the accuracy of ±0.2°C. Participators' skin-surface temperature ranged from 36.5°C to 36.7°C. The effect of temperature on participators' impedance was assumed to be negligible in this study for the reason that the skin-surface temperature difference among participators was very small.

The ICC is a measure that can be used to quantify the reproducibility of a variable. It is also a measure of the homogeneity within groups of replicate measurements relative to the total variation between groups. It is suggested that ICC values above 0.75 are indicative of good reliability [30]. Moreover, for many clinical measurements, reliability should exceed 0.90 to ensure reasonable validity [30]. In this study all the ICC(3,1) measurements exceeded 0.90. This suggests they have exceeded the threshold for both good reliability and reasonable validity.

**Figure 7:**
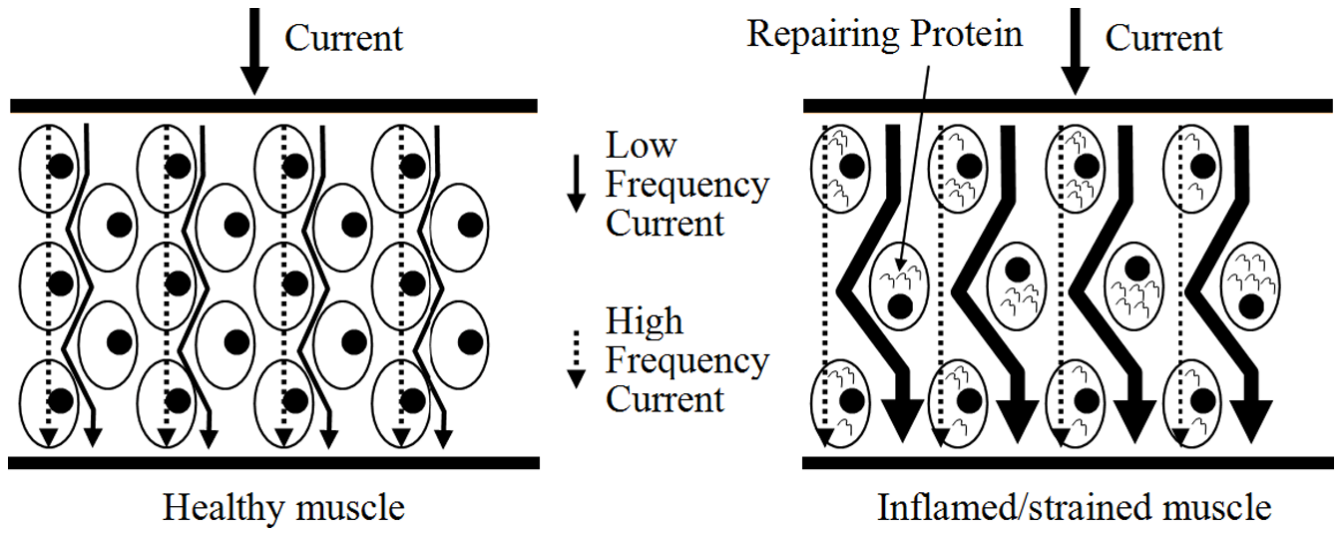
Currents flowing through muscles at different conditions. Schematic diagram shows the route of low and high frequency currents running through a healthy and an inflamed/strained muscle. According to Equation 2, the impedance of a muscle as frequency closed to zero is equal to its extracellular resistance (R_e_). This means that low frequency current primarily runs through the extracellular medium but not the intracellular medium. For inflamed/strained muscle, it has enlarged extracellular gaps because of the local swelling. Therefore, low frequency current has a wider extracellular gap to flow and this result in the reduction in the R_e_.

LBP is often caused by muscle inflammation and strain. It can be marked by localized swelling in the immediate area. Our findings showed that the four studying parameters (Z, R_e_, R_i_, and R_e_/R_i_) have the ability to distinguish the LPM of LBP patients and healthy subjects. Results showed that Z of LBP patient's LPM was significantly smaller (p<0.05) than that of healthy subject's LPM at 0.02 kHz. Also, R_e_ of LBP patient's LPM was significantly smaller (p<0.05) than that of healthy subjects. The relatively lower Z and R_e_ in LBP patient's LPM might be due to the muscle inflammation which commonly results in local swelling (i.e. more blood and interstitial fluid at that region for the facilitation of muscle repair). A possible explanation for the changes observed is as follows. As shown in Equation 2, current at low frequency (<1 kHz) primarily flows around the muscle cell without being able to directly go through the muscle cell (Figure 7). For healthy subjects, their muscle cells are well packed and closed to each other. Hence, low frequency (0.02 kHz in this study) current has very restricted intercellular pathways to run and this result in a high resistance. But, for acute LBP patients, their muscles normally have a phenomenon of inflammation and strain, which have adverse effects on resistance. Inflamed/strained muscles have enlarged extracellular gaps because of the local swelling and this would be expected to decrease resistance. Therefore, acute LBP patients normally have a relatively lower Z and R_e_ in their LPM. On the other hand, current at high frequency (>1 kHz) flows around and penetrates the muscle cell at the same time (Figure 7). For acute LBP patients, their inflamed/strained muscles are under repair and hence many repairing proteins are synthesized inside the muscle cells. This results in a dramatically increase of intracellular resistance and this phenomenon has been further proved in Figure 5. Owing to the increase of intracellular resistance of inflamed/strained muscles and based on Equation 3, inflamed/strained muscles will have a higher Z as compared to healthy muscles if the Z is measured at high frequency (>1 kHz). Therefore, as shown in Figure 3, Z of LBP patient's LPM was found to be significantly (p<0.05) higher than that of healthy subject's LPM at 25.02, 50.02, 1000.02, 3000.02 and 5000.02 kHz. Also, R_i_ of LBP patient's LPM was found to be significantly greater (p<0.05) than that of healthy subjects (Figure 5).

Self-normalization idea has been utilized in the studying parameter of R_e_/R_i_. The use of self-normalization aims to keep away from the large subject-to-subject differences in LPM during data analysis. Therefore, this allows the electrical properties of LPM to be studied without the nonuniformities introduced by patient-to-patient variation. Hence, R_e_/R_i_ is more independent and trustable on distinguishing the LPM of LBP patients and healthy subjects. As shown in Figure 6, it was observed that R_e_/R_i_ of LBP patient's LPM was significantly smaller (p<0.05) than that of healthy subjects.

EIM have several advantages such as low-cost, providing instant result, and little training requirement. Besides, provision of instant assessment results can be able to repeat inadequate assessment immediately. Therefore EIM can be easily used in primary care.

## Conclusions

Significant separation of LPM of LBP patients and healthy subjects could be attained using the measurement parameter of Z, R_e_, R_i_ and R_e_/R_i_. The ratio R_e_/R_i_ is an independent and trustable measurement parameter for distinguishing the LPM of LBP patients and healthy subjects. All measurements had good reliability with ICC>0.95. This method can be used as a potential screening test with the advantage of providing an immediate result. Also, this method may be used by those with minimal training in the setting of primary care or in the developing countries.
